# Fungal Pathogens in Grasslands

**DOI:** 10.3389/fcimb.2021.695087

**Published:** 2021-08-09

**Authors:** Anuruddha Karunarathna, Saowaluck Tibpromma, Ruvishika S. Jayawardena, Chandrika Nanayakkara, Suhail Asad, Jianchu Xu, Kevin D. Hyde, Samantha C. Karunarathna, Steven L. Stephenson, Saisamorn Lumyong, Jaturong Kumla

**Affiliations:** ^1^Centre for Mountain Futures, Kunming Institute of Botany, Kunming, China; ^2^Department of Entomology and Plant Pathology, Faculty of Agriculture, Chiang Mai University, Chiang Mai, Thailand; ^3^Center of Excellence in Fungal Research, Mae Fah Luang University, Chiang Rai, Thailand; ^4^CIFOR-ICRAF China Program, World Agroforestry (ICRAF), Kunming, China; ^5^School of Science, Mae Fah Luang University, Chiang Rai, Thailand; ^6^Department of Plant Sciences, University of Colombo, Colombo, Sri Lanka; ^7^State Key Laboratory for Conservation and Utilization of Bio-Resources in Yunnan, Yunnan Agricultural University, Kunming, China; ^8^Department of Biological Sciences, University of Arkansas, Fayetteville, AR, United States; ^9^Research Center of Microbial Diversity and Sustainable Utilization, Faculty of Science, Chiang Mai University, Chiang Mai, Thailand; ^10^Department of Biology, Faculty of Science, Chiang Mai University, Chiang Mai, Thailand; ^11^Academy of Science, The Royal Society of Thailand, Bangkok, Thailand

**Keywords:** Ascomycetes, foliar diseases, graminicolous fungi, grassland ecology, human and plant disease, phytopathogens, soil-borne diseases

## Abstract

Grasslands are major primary producers and function as major components of important watersheds. Although a concise definition of grasslands cannot be given using a physiognomic or structural approach, grasslands can be described as vegetation communities experiencing periodical droughts and with canopies dominated by grasses and grass-like plants. Grasslands have a cosmopolitan distribution except for the Antarctic region. Fungal interactions with grasses can be pathogenic or symbiotic. Herbivorous mammals, insects, other grassland animals, and fungal pathogens are known to play important roles in maintaining the biomass and biodiversity of grasslands. Although most pathogenicity studies on the members of Poaceae have been focused on economically important crops, the plant-fungal pathogenic interactions involved can extend to the full range of ecological circumstances that exist in nature. Hence, it is important to delineate the fungal pathogen communities and their interactions in man-made monoculture systems and highly diverse natural ecosystems. A better understanding of the key fungal players can be achieved by combining modern techniques such as next-generation sequencing (NGS) together with studies involving classic phytopathology, taxonomy, and phylogeny. It is of utmost importance to develop experimental designs that account for the ecological complexity of the relationships between grasses and fungi, both above and below ground. In grasslands, loss in species diversity increases interactions such as herbivory, mutualism, predation or infectious disease transmission. Host species density and the presence of heterospecific host species, also affect the disease dynamics in grasslands. Many studies have shown that lower species diversity increases the severity as well as the transmission rate of fungal diseases. Moreover, communities that were once highly diverse but have experienced decreased species richness and dominancy have also shown higher pathogenicity load due to the relaxed competition, although this effect is lower in natural communities. This review addresses the taxonomy, phylogeny, and ecology of grassland fungal pathogens and their interactions in grassland ecosystems.

## Introduction

There is no concise and unambiguous definition for grasslands (Gibson, 2009). The definition of a grassland could be based on the absence of specific vegetation features (Milner and Hughes, 1969) when using a physiognomic or structural approach (Bazzaz and Parrish, 1982; Sims, 1988). A more promising definition, however, was given by Risser (1988), who indicated that grasslands are “types of vegetation that are subject to periodic drought, that have a canopy dominated by grass and grass-like species, and that grow where there are fewer than 10 to 15 trees per hectare”. Grasslands are distributed throughout the world’s land area except on the continent of Antarctica (Gibson, 2009). Based on the “The Pilot Analysis of Global Ecosystems (PAGE)” classification, grasslands cover 52,544,000 km^2^ or 40.5% of the world’s land mass (excluding urban areas according to night time lights) (White et al., 2000; Kassam, 2002). According to the PAGE classification, grasslands cover more land area than the other major vegetation cover types (White et al., 2000). For example, forests cover 28.97 × 10^6^ km^2^ and agriculture covers 36.23 × 10^6^ km^2^ (White et al., 2000). Furthermore, grasslands are the second largest land type inhabited by humans (nearly 800 × 10^6^ people), second only to agricultural land, which holds 2.8 × 10^9^ people according to 1995 estimates (White et al., 2000; Gibson, 2009). Grasslands also occupy comparatively large areas of many major watersheds in the world (Gibson, 2009).

The effect of species diversity on the productivity of a community has been explained by two mechanisms. They are: (i) the sampling effects, which state that the probability of finding key-trait species in a community is reduced due to lower species richness (Aarssen, 1997; Huston, 1997; Tilman et al., 1997) and (ii) the niche complementarity hypothesis, which states that less diverse communities with competing species utilize resources incompletely (Naeem et al., 1994; Tilman et al., 1996; Hector et al., 1999). Furthermore, the loss of interactive competition and loss of species diversity have been shown to increase mutualism, predation, herbivory, and infectious disease transmission (Bond, 1993; McNaughton, 1994; Chapin et al., 1997; Chapin et al., 2000). Mitchell et al. (2002) tested the long-standing hypothesis (Elton, 1958; van der Plank, 1963) that the lower diversity of plant species increases the severity of diseases, focusing mainly on specific pathogens. Several studies have previously suggested that this hypothesis applies only to a small number of plant species and genotypes (Garrett and Mundt, 1999; Zhu et al., 2000). The relaxed interspecific competition due to the decreased plant species richness has been shown to increase the abundance of one or more species existing in a local community, which typically also increases the abundance of one or more host species for specialist pathogens (Aarssen, 1997; Huston, 1997; Tilman et al., 1997). The basic mechanism of the diversity-disease hypothesis is that a decreasing number of plant species allows for an increased local abundance of other singular species, which then facilitates the spread of diseases specific to that species within the community (Burdon and Chilvers, 1976; Knops et al., 1999; Chapin et al., 2000). The extent of the differences among different species is correlated with the ecological effects of species diversity (Tilman and Lehman, 2014); hence, the susceptibility of different species for a particular disease must vary. Thus, the local mechanisms of the diversity-disease hypothesis vary highly, as the host abundance depends on numerous biotic and abiotic factors in communities (Mitchell et al., 2002). Apart from host abundance, many other factors such as microclimate and the competitive ability of host plants also influence the disease level in ecosystems with decreasing diversity (Boudreau and Mundt, 1992; Boudreau and Mundt, 1994; Zhu et al., 2000; Boudreau and Mundt, 2018).

Most studies examining the diversity-disease hypothesis have been focused on agriculture or silviculture (Mitchell et al., 2002). However, studies by Kranz (1990) revealed that natural, communities with higher diversity did not necessarily have low disease levels, whereby the diversity of forest and pasture communities was higher while in meadow and agricultural field communities it was lower (Kranz, 1990). As such, species diversity is related to species composition, microclimates, and many other factors (Kranz, 1990; Mitchell et al., 2002). Agronomic intercropping, with increased species diversity, subsequently decreases the prevalence of diseases (Mitchell et al., 2002), especially fungal diseases. Experimental studies on the diversity-disease hypothesis with regards to intercropping are fewer (Boudreau and Mundt, 2018), but intercropping can change the microclimate and the competitive nature of the crops and all these factors together can either increase or decrease disease severity (Boudreau and Mundt, 1992; Boudreau and Mundt, 1994; Zhu et al., 2000; Boudreau and Mundt, 2018).

It has been shown that cultivating a variety mixture or multiline with multiple genotypes of one crop reduced the severity of airborne fungal diseases when compared with its intercrop cultivation (Wolfe, 1985; Finckh and Wolfe, 1997; Garrett and Mundt, 1999; Zhu et al., 2000; Boudreau and Mundt, 2018). In agricultural systems, the main mechanism of reducing the spread of the disease is through reducing the host abundance (Burdon and Chilvers, 1977; Burdon and Chilvers, 1982; Chin and Wolfe, 1984; Wolfe, 1985; Alexander et al., 1986; Burdon, 1987; Garrett and Mundt, 1999; Zhu et al., 2000; Boudreau and Mundt, 2018). We can overlook highly diverse natural grassland ecosystems through the modern knowledge of agricultural multiline even though natural grasslands are more complex than the agricultural multiline.

Disease susceptibility and the dominant species present are two aggregated characteristics of a community that can influence the spread and severity of a disease (Mitchell et al., 2002). Mitchell et al. (2002) tested several hypotheses relating to disease levels in a community and found that (1) the loss of less susceptible species in a community increased the disease levels within the community more than the loss of highly susceptible species, (2) the loss of less susceptible species from a plant community increased the disease levels in that community, and (3) the loss of the dominant species increased the prevalence of species-specific pathogens more than the loss of rare species, when all other conditions were equal. However, in certain instances, the most abundant species can be more susceptible to disease, and in such cases the above hypotheses are not valid and the effect of losing the dominant, highly susceptible species is countervailing (Arneberg et al., 1998).

Grasslands are highly diverse ecosystems with many interactions. Plant communities in grasslands are comprised mainly of diverse species of grasses, including genotypes of the same species, and various dicots. However, the major part of the community is represented by grasses. Population structure of grasslands is crucial for the productivity as well as the resource utilization of grasslands. However, pathogens in grasslands play a crucial role in the productivity of these communities. Herein we investigate how fungal pathogens presence affect the dynamics of grasslands. Population dynamics of fungal pathogens are highly influenced by the diversity and the population structure of grassland is in question. Furthermore, the dynamics of specialist pathogens and generalist pathogens are contrasting. Increased host diversity of grasslands increases generalist pathogens while reducing specialist pathogens. However, this may be highly vary based on the many biotic and abiotic factors in grasslands. Hence, interactions between pathogens and their host in natural grasslands are complex.

This review brings together a vast amount of information on fungal pathogens in grasslands and then discusses their role within and effects on grassland ecosystems. This also describes the pathogenic fungal communities in grasslands and discusses well-studied pathogenic fungal species reported on host grasses.

## Pathogenic Fungal Communities in Grasslands and Their Interactions

Pathogenic fungi significantly affect the population biology of grasses and their contribution to plant communities by affecting the physiology and chemical composition of those grasses (Gibson, 2009). This in turn affects the population ecology of grassland communities and ecosystems, especially when they affect the dominant species of those communities (Burdon et al., 2006). However, the pathogenic effects of fungi cannot be studied by considering only the fungi themselves; they manifest their effects *via* complex species interactions (Ampt et al., 2019).

Ecologists have observed that two main aspects drive the interactions between plant species and their natural enemies in natural ecosystems—host-specificity and density-dependency (Janzen, 1970; Connell, 1971; Bever et al., 2015). According to the “pathogen hypothesis”, the negative effects from a pathogen on a host must either be species-specific affecting certain species only, or a generalist which can have negative effects on multiple host species (Hersh et al., 2012; Ampt et al., 2019). Mommer et al. (2018) studied monocultures of four grass species and four forbs found in natural grasslands. In this study, the Internal transcribed spacer (ITS) based Next-Generation Sequencing (NGS) analysis of soil-borne fungal species showed a clear difference in the host specificities of fungal communities among grasses and forbs. In addition, host specificity has been apparent in plant-soil feedback studies, where the same species or the functional group, has been grown and the plant growth was reduced (Petermann et al., 2008; Mangan et al., 2010; Hendriks et al., 2013; Cortois et al., 2016). It is important to use NGS complemented analysis in conjunction with traditional pathology knowledge to gain a proper knowledge of the interactions between soil-borne fungal pathogens and their plant hosts (Mommer et al., 2018). An insight into the host specificity of soil-borne fungal pathogens in natural grasslands has been provided by Klironomos (2002). Klironomos (2002) isolated fungi from roots of several rare plants found in Canadian meadows, which were later identified as species of *Cylindrocarpon*, *Fusarium*, and *Verticillium*. Although identification of these fungi was only to the level of the genus, in the ecological context host specificity was clearly demonstrated upon inoculation to the field as there was a reduction in plant growth as a result of fungi isolated from conspecific roots but not from heterospecific roots (Klironomos, 2002). Consequently, this demonstrated that those fungi had only a negative impact on their preferred host (Gibson, 2009).

Biodiversity experiments in natural grasslands have also demonstrated pathogen dilution in foliar (Knops et al., 1999; Mitchell et al., 2002; Rottstock et al., 2014) and soil-borne fungi (Mommer et al., 2018). Rottstock et al. (2014) showed that foliar pathogen incidence and severity were reduced with increased species diversity. However, Ampt et al. (2019) suggested that the results might be due to a sampling error. Another study examined the effect of plant density on soil-borne disease dynamics in relation to seedling mortality in agricultural systems (Otten et al., 2004; Otten et al., 2005; Hiddink, 2008). During experiments on Wageningen grassland biodiversity (Van Ruijven et al., 2003; Van Ruijven and Berendse, 2005; Van Ruijven and Berendse, 2009; Cong et al., 2014), nearly 50% of the variation in fungal community composition could be explained by the density of the below ground host components. Hence, host density has a major impact on the fungal community. Moreover, above ground fungal pathogens play a major role in biomass of the grasslands (Cappelli et al., 2020). The recent studies by Cappelli et al. (2020) showed that the infection incidence is highly affected by trade-off between plant growths and defines with the consideration of diverse grasslands and generalist pathogens as the generalist pathogens can spread out easily within a highly diverse grassland.

Although host specificity and host density both play major roles in the disease dynamics within a community, heterospecific neighbors also have an impact (Otten et al., 2005; Eppinga et al., 2006). Increased heterospecific neighbor plants, or the host diversity, reduce the host density of the specialist pathogens while increasing the density of generalist pathogens (Petermann et al., 2008). This evidence was observed in agricultural intercropping, where the presence of maize reduced Phytophthora blight severity and spread in pepper plants (Yang et al., 2014). The heterospecific neighbor effect can be explained in two ways by considering the below ground disease dynamics (Ampt et al., 2019). First, there are direct neighbor effects occurring through plant traits and root exudates. Second, there are indirect neighbor effects through the root microbiome (Ampt et al., 2019). Heterospecific neighbors can either act as barriers or vectors for diseases (Otten et al., 2005; Ampt et al., 2019). Host plants surrounded by non-host plants are less likely to become infected, and hosts that are surrounded by the hetero-specific hosts or symptomless hosts are susceptible to becoming infected (Malcolm et al., 2013). Furthermore, phylogenetically distant hosts are less likely to infect other hosts, and thereby plant communities with phylogenetically diverse hosts are less likely to spread disease within themselves (Gilbert and Webb, 2007; Haas et al., 2011; Wehner et al., 2014; Gilbert and Parker, 2016). The root system of different species may also either enhance or reduce disease transmission by physical or chemical means (Newsham et al., 1995). Intercropping of maize and pepper reduced the spread of Phytophthora blight due to the higher degree of root intermingling (Yang et al., 2014). Similarly, the dense and tightly intermingled roots of natural grasslands reduce the spread of disease (Kesanakurti et al., 2011; Ravenek et al., 2014; Frank et al., 2015). Moreover, complex below ground chemical composition and chemical communication has an impact on disease dispersal as well, but these mechanisms are not yet well explained (Ampt et al., 2019). Indirect neighbor effects *via* the root microbiome have been shown in natural grasslands inhabited by species of *Streptomyces* (Bakker et al., 2013; LeBlanc et al., 2015).

In natural grasslands, hosts, fungi, and pathogens are highly correlated. Below-ground host components shape total fungal composition while above-ground pathogens shape the biomass of grasslands. Moreover, generalist pathogens are highly dispersed in grasslands characterized with rich diversity. Hence, in grasslands, disease incidence highly relies on the plant growth, diversity of grasslands, and generalist pathogens in grasslands. Host specificity, host density, and host diversity play huge roles in the dynamics of grasslands. Heterospecific neighbors disturb the spread of specialist pathogens in grasslands. However, the presence of heterospecific neighbors increases the spread of generalist pathogens. However, heterospecific hosts can be either barriers or vectors if the host is symptomless. The close phylogenetic relatedness of grasses can increase disease spread among grasses with shared traits. Heterospecific neighbors can affect the spread of diseases through plant traits or root microbiomes. However, the tightly intermingled roots of heterospecific neighbors reduce specialist pathogens in grasslands.

## Soil-Borne Fungal Pathogens in Grasslands

The majority of studies on grassland pathogens have been focused on above ground systems and monocultures for a few primary reasons (Ampt et al., 2019). The air-borne dispersal and pest-based dispersal of above ground pathogens attributes to fast spread of the pathogen and can be detected much more easily, especially under epidemics (Mitchell et al., 2002; Rottstock et al., 2014). Particularly in monoculture systems, the diseased areas can be visually seen as gaps in cultivation drawing immediate attention. Furthermore, much of the research attention in this area has been focused on monocultures, as they are often a part of economically important agricultural systems (Ampt et al., 2019).

Plant species richness has been shown to increase plant productivity, and soil-borne fungal pathogens play an important role in plant productivity (Maron et al., 2011; Schnitzer et al., 2011; Cardinale et al., 2012; Mommer et al., 2018). Experiments on how biodiversity loss affects ecosystem functioning in experimental grasslands has shown that monocultures perform less well than polycultures in measures of plant productivity (Tilman et al., 1997; Hector et al., 1999; Tilman et al., 2001; Van Ruijven and Berendse, 2005). However, the mechanism responsible for this scenario is not clear (Cardinale et al., 2012). The most promising strategy for explaining the difference in plant productivity has been by focusing on plant-plant interactions, resource partitioning, and facilitation between plants (Mueller et al., 2013; Ravenek et al., 2014; Wright et al., 2017; Jesch et al., 2018). Furthermore, interactions between the plants and soil biota are important contributor for positive biodiversity effects. The main hypothesis regarding the effect of soil-borne pathogens is that their negative impact is stronger in plant monocultures and weaker in mixed-plant ecosystems (Maron et al., 2011; Schnitzer et al., 2011; de Kroon et al., 2012; Mommer et al., 2018). Ecologists have investigated the “pathogen hypothesis” using a black box method, such as comparing plant growth on soil with and without the soil biota (Kos et al., 2013; Mccarthy-Neumann and Nez, 2013; Hendriks et al., 2015; Cortois et al., 2016; Wubs and Bezemer, 2018). Schnitzer et al. (2011) grew plant communities in both field and sterile soil. The result showed that on sterilized soil, the positive effects of plant species richness on plant productivity disappeared. Sterilization enhanced the productivity at lower biodiversity but did not affect biodiversity of soil with high plant species richness. Again, Schnitzer et al. (2011) re-inoculated the sterilized soil using a soil wash containing soil-borne fungi and observed that the productivity for the lower diversity group was reduced. This emphasized the importance of soil-borne fungi on biodiversity patterns. Maron et al. (2011) experimented on the relationship between plant diversity and above-ground plant biomass in fungicide treated and non-treated soils. Application of systemic fungicides thiophanate ethyl and mefenoxam adversely affected this relationship indicating the positive effect exerted by soil fungi on plant diversity and productivity (Maron et al., 2011). The experiments of Maron et al. (2011) and Schnitzer et al. (2011) demonstrated the importance of host specific microorganisms in determining the diversity-productivity relationship. Hence it can be stated that a deep understanding of the roles played by soil-borne fungi in grasslands is essential in defining the exact roles of grassland associated fungi.

The most studied soil-borne pathogens of grasses are the ones that have been known to cause severe diseases for many hosts, especially for agriculturally important crops (Ampt et al., 2019). For example, *Fusarium oxysporum* causes Fusarium wilt on nearly 100 monocot and dicot hosts (Michielse and Rep, 2009). Soil-borne fungal diseases have been reported from commercially grown medicinal herbs and turf grass species found in natural grasslands (Smiley et al., 2005). Furthermore, soil-borne fungal diseases have also been identified in commercial monocultures of dicots found in grasslands (Gaetán et al., 2004). However, this knowledge has not been found to provide very much insight into fungal-plant interactions of highly diverse grassland ecosystems (Ampt et al., 2019).

Natural grasslands are diverse ecosystems housing a variety of monocot and dicot plants (Herben et al., 1993; Van Der and Sykes, 1993). Hence, grassland ecosystems are highly static yet show a wide variety of spatiotemporal dynamics (Herben et al., 1993; Van Der and Sykes, 1993). Soil-borne pathogens play a major role in these spatiotemporal dynamics (Olff et al., 2000). Soil-borne fungal diseases in natural grasslands are rarely observed, either because the disease incidence is low in higher diversity communities or other species replace the poorly performing species before they have a significant effect (Gilbert, 2002; Burdon et al., 2006; Alexander, 2010). Soil-borne pathogens in grasslands are highly diverse due to the overall high species diversity and the genotype diversity for a given species (Termorshuizen, 2014; Dassen et al., 2017; Yang et al., 2017; Bach et al., 2018). Although plant root systems in grasslands are colonized by a wide variety of fungi, only a few have been isolated and had their pathogenicity thoroughly examined through Koch’s postulates (Vandenkoornhuyse et al., 2002). Mills and Bever (1998) isolated five species of *Pythium* from two types of perennial grass, *Danthonia spicata* and *Panicum sphaerocarpon*, from a 50-year-old grassland. They reintroduced four species of *Pythium*, and it reduced the biomass of *D. spicata* and *P. sphaerocarpon*, while *Anthoxanthum odoratum* (Poaceae) and *Plantago lanceolata* (Plantaginaceae) were not adversely affected by the four reintroduced species of *Pythium*. In the most recent study of grassland biodiversity by Mommer et al. (2018), the authors isolated 27 species of fungi from symptomatic roots of *Anthoxanthum odoratum* (Poaceae) and the forb *Leucanthemum vulgare* (Asteraceae). Among the isolates, *Magnaporthiopsis panicorum* and *Paraphoma chrysanthemicola* caused host specific adverse effects on seedlings (Mommer et al., 2018). Hence, root inhabiting pathogens can cause a considerable loss on the biomass of natural grasslands (Ampt et al., 2019).

Although the mechanisms of soil-borne plant pathogenic fungi affecting the spatiotemporal dynamics of natural grassland ecosystems are not well known, three main pieces of experimental based evidence have been identified (Ampt et al., 2019). First, next generation sequencing (NGS) has been used to identify a very high diversity of soil-borne fungal community growing on roots of grassland plants. Second, some soil-borne fungi have been observed to negatively affect plant growth in bioassays. Third, the relationship between plant diversity and plant productivity is affected by the sterilization of the soil and by application of fungicides (Ampt et al., 2019). However, the interactions of soil-borne fungi and their effects on plants within the larger context of biodiversity are yet to be discovered.

In natural grasslands, strategies that account for different productivities are plant-plant interactions, resource partitioning, and facilitation between plants. In species-rich grasslands, soil-borne fungi play an immense role in increasing the productivity of grasslands. However, in less-diverse grasslands, soil-borne fungi reduce productivity. Hence, stable grasslands are in proper equilibrium with host species and soil-borne fungal communities. In highly diverse grasslands soil-borne fungal diseases are rarely observed due to the low disease incidence and the natural replacement of poorly performing species. However, in general, high species or genotypic diversity of grasses leads to high diversity of soil-borne pathogens, although disease expression depends on the traits of host plants. Furthermore, the monocots and dicots in grasslands share the same soil-borne pathogens.

The studies on above ground fungal pathogens of grasses mentioned above were primarily studies based on agricultural monocultures. Above ground fungal pathogens in complex natural grassland ecosystems are much less studied. In the following sections, several well-studied fungal pathogens found in agricultural monocultures will be discussed.

## Selected Fungal Pathogens on Grasses

There are many studies of pathogens on grasses. Although the pathogen-related studies are mainly focused on economically important monocultures. They provide invaluable information about aspects of populations involved as well as their genomes. As such, in this section we describe several studies on selected well studied pathogens to understand how important it is to carry out a proper study on pathogenic fungal population in natural grasslands. Herein we are describing four well studied species: *Bipolaris sorokiniana, Colletotrichum graminicola, Fusarium graminearum*, and *Pyrenophora tritici-repentis.* Each species was selected in order to explain significant characteristics of fungal pathogens relevant to the Poaceae. The race structure of *Pyrenophora tritici-repentis* and its relatedness to economically important crops as well as certain instances where it is relevant to the natural grassland systems along with data on genomes relevant to the pathogenicity have been intensively studied*. Fusarium graminearum* has been well studied for its mycotoxin production and its involvement with pathogenicity. *Bipolaris sorokiniana* is a well studied pathogen noted for its variations in pathogenicity, DNA polymorphism, and adaptability. *Colletotrichum graminicola* has been well studied for the infection mechanism and involvement of proteins in inducing diseases. Some of the common details of those pathogens are given in **Table 1**.

**Table 1:** Host, distribution factors and overwintering stages of selected fungal pathogens.

Pathogen	Disease	Host	Distribution factors	Overwintering stages	References
*Pyrenophora tritici-repentis*	Tan spot	Cereal grasses (*Avena sativa*, *Bromus* spp., *Dactylis glomerata*, *Hordeum vulgare*, Secale cereale, *Triticum aestivum* and *T*. *turgidum*), noncereal grasses including native prairie grasses and other species of grasses	Humidity induces the conidial production of *Ptr* and conidia disperse by wind	Saprophytic stage on wheat stubble as pseudothecia	Hosford, 1971; Krupinsky, 1986; Krupinsky, 1992a; Schilder and Bergstrom, 1992; Ali and Francl, 2003; Bockus et al., 2010; Ciuffetti et al., 2014
*Fusarium graminearum*	Head blight	Wheat, barley, oats, and many other small grain cereal crops	Ascospores and macroconidia can be disseminated by wind, rain, and insects to host plants and deposited on or inside of spike tissues. anthers are suggested as vulnerable sites for primary infection	Perithecia at spring	Sutton, 1982; Guo and Ma, 2014
	Ear rot and stalk rot	Maize			
*Bipolaris sorokiniana*	foliar spot blotch, root rot, and black points	Cereal crops such as wheat (*Triticum aestivum*) and barley (*Hordeum vulgare*)	Contaminated soil and contaminated plant debries	Dormant mycelium or conidia in infected plant tissues, thatch, and in plant debris	Kumar et al., 2002; Dai et al., 2011; Dicklow 2011; Kang et al., 2021;
*Colletotrichum graminicola*	Anthracnose, causing stalk rot and seedling blight	Maize	Wind and raindrop splashes	Saprophytically on dead plant material to over winter (Nordzieke et al., 2019)	Bergstrom and Nicholson, 1999; Nordzieke et al., 2019

### Pyrenophora tritici-repentis

*Pyrenophora tritici-repentis* (Died.) Drechsler is one of the most widely studied pleosporalean fungi that causes an economically important disease (Ciuffetti et al., 2014) (**Table 1**). From this point onwards, *Pyrenophora tritici-repentis* is abbreviated as *Ptr. Pyrenophora tritici-repentis* shows the homothallic nature of sexual reproduction with the presence of MAT genes MAT1-1 and MAT1-2 in a single locus (Lepoint et al., 2010).

**Figure 1:**
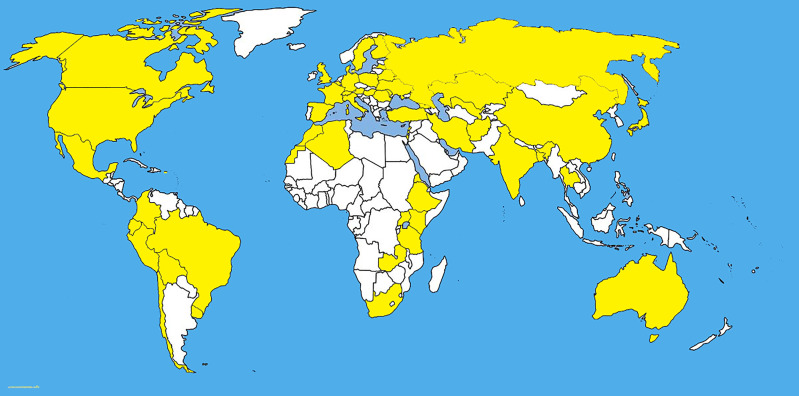
The distribution map of *Pyrenophora tritici-repentis*. The areas where infection have been reported are indicated in yellow (www.cabi.org/isc. Accessed on 25/03/2021).

**Figure 2:**
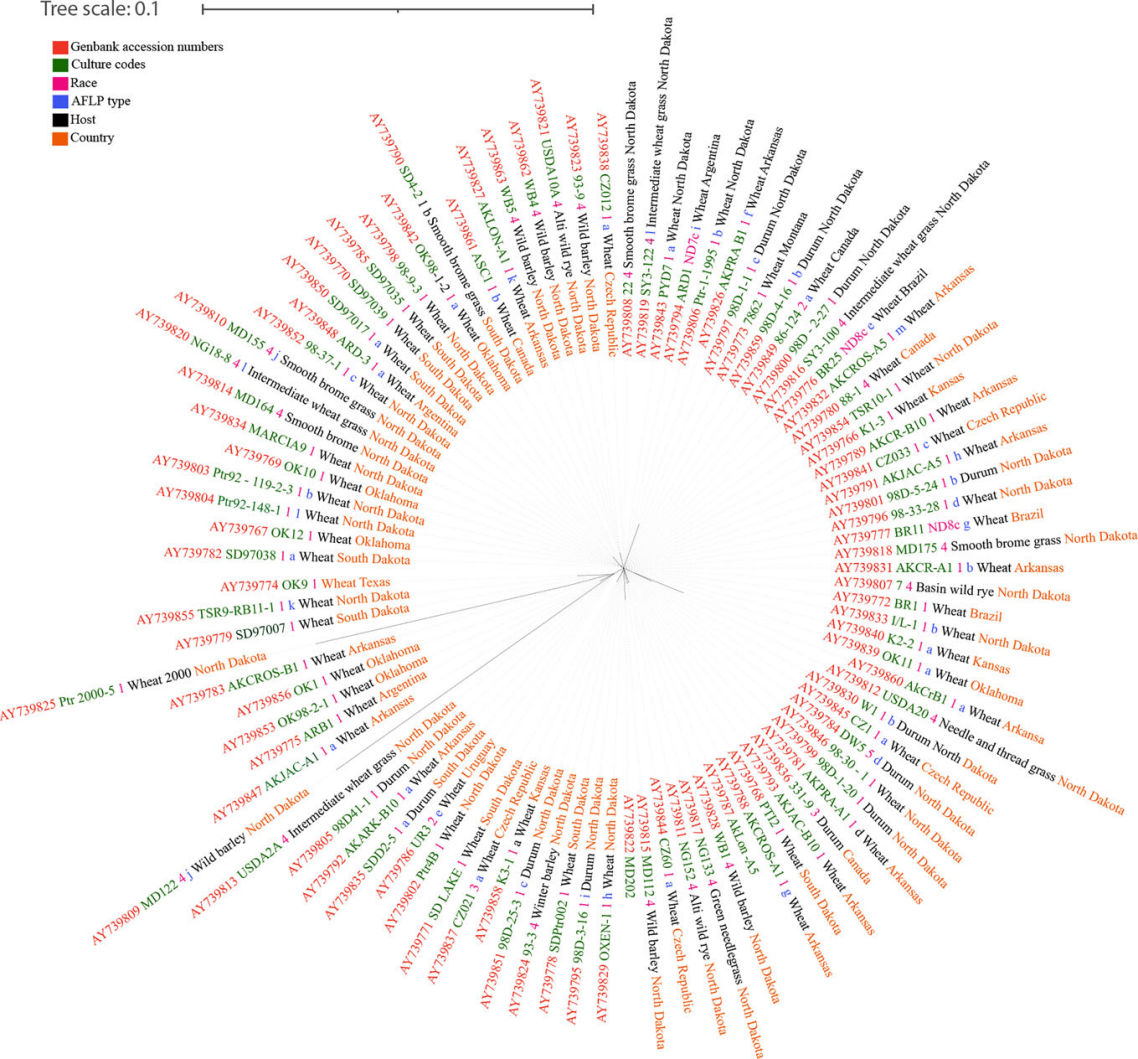
The unrooted RAxML bipartion unrooted phylogenetic tree of *Pyrenophora tritici-repentis* based on Friesen et al. (2005).

*Pyrenophora tritici-repentis* has been isolated from cereal grasses (*Avena sativa*, *Bromus* spp., *Dactylis glomerata*, *Hordeum vulgare*, *Secale cereale*, *Triticum aestivum*, and *T. turgidum*) along with noncereal grasses including native prairie grasses and other species of grasses (Hosford, 1971; Krupinsky, 1986; Krupinsky, 1992a; Ali and Francl, 2003; Bockus et al., 2010) (**Figures 1**, **2**). With the studies of monocultures, it has been found that *Ptr* can overwinter saprophytically as a sexual stage with the asexual stage appearing with the growing stage of the host (Krupinsky, 1992b). Humidity induces the conidial production of *Ptr* and conidia disperse by wind (Schilder and Bergstrom, 1992). The latter is responsible for the multiple inoculations of all stages of the host which defines the symptom severity on mature leaves. Furthermore, the conidia remain viable up to three years inside the infected seeds (Schilder and Bergstrom, 1995; Bergstrom and Schilder, 1998).

The studies of *Ptr* have been focused primarily on tan spot disease of wheat (Ciuffetti et al., 2014). Based on Hosford (1971), the resistance to tan spot differs for different genotypes of wheat. Early knowledge of the physiological specializations of tan spot disease was based on quantitative parameters (Ciuffetti et al., 2014). Additional qualitative studies of tan spot symptoms revealed that necrosis and chlorosis are the most reliable signs to define the physiologic specialization of *Ptr* with the host (Lamari and Bernier, 1989a; Lamari, 1991). Accordingly, Lamari and Bernier (1989a), recognized four pathotypes based on the presence or absence of chlorosis and necrosis on the host tissues. These are pathotype 1 (necrosis+ chlorosis+), pathotype 2 (nec+ chl-), pathotype 3 (nec- chl+), and pathotype 4 (nec- chl-) (Ciuffetti et al., 2014). Later, a race based classification was introduced to characterize pathogenic diversity of *Ptr* (Lamari et al., 1995; Lamari and Strelkov, 2010).

The population studies of *Ptr* have focused primarily on agricultural monocultures and little information is avaiable on natural grasslands. Consequently, herein, we discuss the population and race structure of *Ptr* based on agricultural monocultures. The race structure of *Ptr* has been characterized mainly on the basis of spore inoculation and the host-selective toxins (HSTs) produced on the three wheat genotypes: ‘Glenlea’, 6B365 and 6B662 (Strelkov and Lamari, 2003; Lamari and Strelkov, 2010). Three HSTs in *Ptr* have been recoginzed thus far. They are Ptr ToxA (syn. Ptr necrosis toxin, Ptr toxin, and ToxA) (Ballance et al., 1989; Tomas, 1990; Tuori et al., 1995; Zhang et al., 1997; Ciuffetti et al., 1998), Ptr ToxB (syn. Ptr chlorosis toxin) (Orolaza et al., 1995; Ciuffetti et al., 1998; Strelkov et al., 1999) and Ptr ToxC (syn. Ptr chlorosis toxin) (Ciuffetti et al., 1998; Effertz et al., 2002). Thus far, eight races of *Ptr* have been identified and are described in **Table 2**. Races 3 and 4 are less frequently found on wheat, although they are more prominent in noncereal grasses in the Great Plains (Ali and Francl, 2003). Furthermore, races 7 and 8 were reported from Algeria (Benslimane et al., 2011). In Australian *Ptr*, *ToxB* gene is absent, while *ToxA* is more ubiquotious. As such, races 1 and/or 2 are present in the continent (Antoni et al., 2010; Moolhuijzen et al., 2019).

**Table 2:** *Ptr* races, structure and distribution.

Race	HSTs	Patho type	Predominant regions
Race 1	ToxA and ToxC	1 and 2	North Africa (Benslimane et al., 2011)
Race 2	ToxA	1 and 2	Great Plains of North America (Lamari et al., 1995; Lamari et al., 1998; Ali and Francl, 2003; Singh, 2007; Aboukhaddour et al., 2013) and the Southern Cone Region of South America (Singh, 2007)
Race 3	ToxC	3	North America (Lamari et al., 1998; Ali and Francl, 2003; Singh, 2007; Aboukhaddour et al., 2013) and the Caucasus region (Lamari et al., 2005)
Race 4	No HTSs	4	North America (Lamari et al., 1995; Lamari et al., 1998; Ali and Francl, 2003; Singh, 2007; Aboukhaddour et al., 2013) and North Africa (Benslimane et al., 2011)
Race 5	ToxB		Algeria (Lamari et al., 1995), the United States (Ali et al., 1999), Canada (Ali et al., 1999), Azerbaijan, and Syria (Lamari et al., 2005) but is rarely found in North America
Race 6	ToxB and ToxC		Algeria (Strelkov et al., 2002)
Race 7	ToxA and ToxB		middle east and Caucasus regions (Lamari et al., 2003; Lamari et al., 2005)
Race 8	ToxA, ToxB, and ToxC		middle east and Caucasus regions (Lamari et al., 2003; Lamari et al., 2005)

Apart from the eight well characterized races of *Ptr*, reports have suggested the presence of more races (Ciuffetti et al., 2014). Proper PCR-based analyses are needed to confirm the presence of *ToxA* and *ToxB* genes (Andrie et al., 2007). There are no molecular tests available for *ToxC*, and classification has to rely on the reaction of the differential line 6B365 (Ciuffetti et al., 2014). Ali et al. (2010) reported Ptr strains which induce necrosis even in the absance of the *ToxA* gene, which was known to be the necrosis-inducing toxin. Benslimane et al. (2011) isolated five strains causing disease on tetraploid but not hexaploid wheat. This study suggested the presence of novel races. Hence, all these facts suggest that even considering the monocultures, defining the races requires more collections and molecular level studies. This confirms the complexity of studying the *Ptr* in natural grasslands and the importance of uncovering novel races to develop a proper understanding on race distribution and population dynamics.

One of the earliest studies on genetic diversity of *Ptr* was an effort to develop an understanding about the relationship between isolates from North America and North Africa using random amplified polymorphic DNA (RAPD) markers (Aung, 2001). In that study, a significant difference was shown between the isolates of race 1 and 2 which produce the necrosis-inducing ToxA and necrosis noninducing races 3, 5, and 6, which do not produce ToxA. Later, the studies by Aboukhaddour et al. (2011), who used simple sequence repeat (SSR) markers, yielded data that agreed with what Aung (2001) had reported, with significant genetic differentiation detected. This differentiation grouped the known eight races of *Ptr* into four distinct populations based on their region of origin (Aboukhaddour et al., 2011). Interestingly, Aboukhaddour et al. (2011) revealed that ToxA nonproducing and ToxA-producing isolates are to be distantly related. Moreover, it was suggested that the host-specificity imposed by the Ptr toxins may lead to differentiation among isolates of *Ptr* (Aboukhaddour et al., 2011). Based on amplified fragment length polymorphism (AFLP) analysis, Friesen et al. (2005) concluded that the *Ptr* population is preferentially outcrossing and that spread of the pathogen is recent or constant, and also cosmopolitan. The disagreement between Aboukhaddour et al. (2011) and Aung (2001) with Friesen et al. (2005) suggests the possibility of the differential origin of the isolates (Ciuffetti et al., 2014). The results obtained by Aboukhaddour et al. (2011) are more reliable regarding the diversity of the entire *Ptr* genome because some of the SSR loci located more than 2 Mb apart even on the same chromosome. A broad study of 12 SSR markers showed moderate to high population differentiation between continents (Gurung et al., 2013). Furthermore, Aung (2001) reported 26% similarity between pathogenic and non-pathogenic isolates, while Aboukhaddour et al. (2011) reported 25%. Mating type locus (MAT) based phylogenetic analysis of *Ptr* races between 1-5 has found that the latter represent two distinct phylogenetic groups where one group is characterized by a higher homogeneity of typical tan spot producing strains, while the second group with a higher heterogeneity, including race 4 with small lesion producing strains from wheat and other hosts (Lepoint et al., 2010). Higher levels of genomic diversity (Lichter et al., 2002; Aboukhaddour et al., 2009) and karyotype polymorphisms between pathogenic and nonpathogenic strains were observed in the chromosome-based characterization of *Ptr* isolates by Lichter et al. (2002). These studies revealed the genetic diversity and the significant genomic difference between pathogenic and non pathogenic *Ptr* diversity among monocultures and in certain cases among natural grasslands.

The interaction between wheat pathogenic *Ptr* and the host is inverse of the classical gene-for-gene system observed in host-biotroph systems (Wolpert et al., 2002; Strelkov and Lamari, 2003; Friesen et al., 2008; Lamari and Strelkov, 2010). Hence, the genetic locus that conditions the HST sensitivity is known as the susceptibility locus (Ciuffetti et al., 2014). The *Tsn1* gene (*Tan spot necrosis*) is sensitive to ToxA. *Tsc1* and *Tsc2* genes (*Tan spot chlorosis*) sensitive for ToxC and ToxB (Ciuffetti et al., 2014). Furthermore, several more genes have been recognized through conidial inoculations which are not associated with HSTs and have been named as ‘Tsr’ (Tan spot resistance) (Singh and Hughes, 2006; Tadesse et al., 2006a; Tadesse et al., 2006b; McIntosh and Yamazaki, 2008; Singh et al., 2008). Toxin sensitivity was shown to be controlled by the *Tsn1* gene (Lamari and Bernier, 1989b; Faris et al., 1996). Sensitivity to ToxA does not always completely define susceptibility alone (Friesen et al., 2003; Cheong et al., 2004; Faris and Friesen, 2005; Chu et al., 2008; Singh et al., 2008; Chu et al., 2010; Faris et al., 2012) but does influence disease severity to varying degrees depending on the genetic background of the host and the toxin compliment of the pathogen (Ciuffetti et al., 2014). The single dominant gene *Tsc2* produces ToxB on *Ptr* race 5 (Orolaza et al., 1995; Friesen and Faris, 2004). The *Tsc1* gene is responsible for ToxC and *Tsc1*–ToxC system is much complex than the previous two connections (Ciuffetti et al., 2014).

Plant and microbe interactions involve diverse signaling mechanisms which in turn cause changes in gene expressions both in the host and the signal-producing microbe. *Pyrenophora tritici-repentis* races have both saprobic and pathogenic forms. The pathogenic form of the *Ptr* is known as a necrotrophic pathogen due to the formation of HSTs (Ciuffetti et al., 2014). The infection process of *Ptr* is more complex than the infection process of general nectrotrophs where, the infection of *Ptr* includes the necrotrophic form and a reduced biotrophic phase (Larez, 1986; Lamari and Bernier, 1989a; Dushnicky et al., 1996; Dushnicky et al., 1998b; Dushnicky et al., 1998a). The infection phase of *Ptr* starts from spore germination and the penetration of the epidermal cells is completed within 24 hrs following the formation of the appressorium. This leads to the formation of cell wall depositions on epidermal cells due to the compatible/susceptible and incompatible/resistant genotypes suggestive to the biotroph interactions. With the susceptible-resistant interaction, the intercellular hyphal growth in mesophyll cells occurs and leads to the chlorosis and necrosis of the tissue exhibiting the characteristic tanspot symptoms (Ciuffetti et al., 2014). In *Ptr*, HSTs are known to be involved in pathogenesis because the HSTs are toxic only for the susceptible hosts but not to resistant plants or nonhosts (Ciuffetti et al., 2014). The purpose of HSTs in *Ptr* is to induce the necrosis to benefit the fungi. The response of ToxA is rapid as initiating the necrosis within nine hours of HST treatment and the signaling events initiated by toxin perception induce the changes in gene expression, which leads to cell death (Kwon et al., 1998; Rasmussen et al., 2004; Manning and Ciuffetti, 2005; Pandelova et al., 2012).

Broad genomic studies carried out for *Ptr* and karyotype analyses of currently well known races from diverse geographic regions have shown a range of sizes and defernet numbers of chromosomes, both between races and within the same race (Lichter et al., 2002; Martinez et al., 2004; Aboukhaddour et al., 2009). Genome sizes were estimated to range from 25.5 to 48 Mb with 8 to 11 chromosomes (Ciuffetti et al., 2014). The correlation between genome size and race has not been well established (Ciuffetti et al., 2014), although nonpathogenic isolates trended toward smaller genome sizes and chromosome numbers (Ciuffetti et al., 2014).

Pathogenic fungi in grasslands play a major role in shaping the dynamics of these communities. *Pyrenophora tritici-repentis* is a good example to show how certain fungal pathogens behave in a host community. Different genotypes of hosts respond differently to pathogens. Different races of the same fungi cause different symptoms or non-symptomatic conditions for the same host. It has been suggested that the host-specificity imposed by the *Ptr* toxins may lead to differentiation among isolates of *Ptr*. The studies of *Ptr* show moderate to high population differentiation between continents and karyotype polymorphisms between pathogenic and nonpathogenic strains were observed. *Pyrenophora tritici-repentis*, HSTs are known to be involved in pathogenesis because the HSTs are toxic only for the susceptible hosts but not to the resistant plants or nonhosts. Natural grasslands contain different host genotypes. Each genotype behaves differently for the same pathogen. Furthermore, through this differentiation, the different genotypes together with non-host plants can occur as heterospecific neighbors and reduce the disease spread. In addition, the same fungal pathogen species can exist as different races. Hence, the presence of a fungal pathogen in a grassland community may not be apparent unless the proper response is given by the host species or the host genotype. However, in this concern, grasslands can reserve certain pathogens inertly without showing pathogenicity in grassland communities.

### Fusarium graminearum

**Figure 3:**
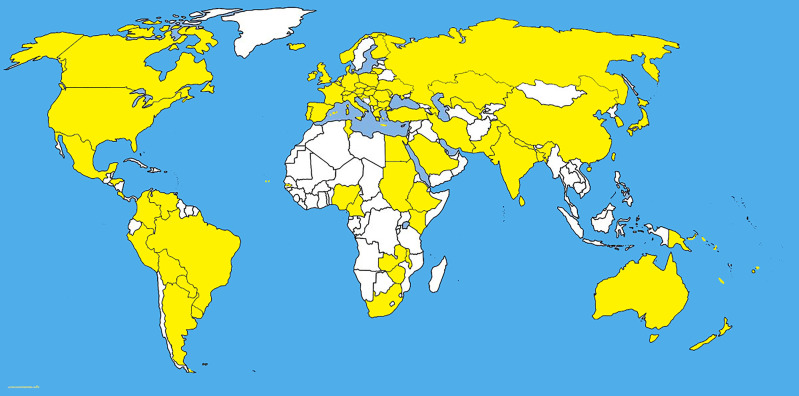
The distribution map of *Fusarium graminearum.* The areas where infection have been reported are indicated in yellow (www.cabi.org/isc. Accessed on 25/03/2021).

*Fusarium graminearum* Schwabe, is an important cosmopolitan pathogen (**Figure 3** and **Table 1**) and produces mycotoxins which are carcinogenic to humans. Like many pathogens, *Fusarium graminearum* is self-fertile and shows facultative outcrossing (Leslie and Summerell, 2007). The primary inoculum of *F. graminearum* causes a head blight infection of wheat and barley (Guo and Ma, 2014).

*Fusarium graminearum* has the unique feature of compartmentalized genome structure (Guo and Ma, 2014). The subtelomeric regions of *F. graminearum* chromosomes are highly polymorphic among different isolates in the population (Guo and Ma, 2014). Furthermore, *F. graminearum* chromosomes also show a high single nucleotide polymorphism (SNP) rate (Guo and Ma, 2014). Those regions are enhanced for genes that are responsible in plant–fungus interactions, including the secretion of proteins and the actual genes expressed (Cuomo et al., 2007). Another remarkable feature in *F. graminearum* is the lack of repetitive sequences (Guo and Ma, 2014).

*Fusarium graminearum* is homothallic (Guo and Ma, 2014). The genome encodes for both the MAT1-1 and the MAT1-2 loci, while other fertile species of *Fusarium* are heterothallic, harboring either MAT1-1 or MAT1-2 and strains having MAT genes that are different and sexually compatible (Guo and Ma, 2014). The homothallic lifestyle of *F. graminearum* might be developed from a self-sterile ancestor. Perithecia serve as a source of inoculum for the stem root rot of pepper caused by *F. solani* f. sp. *piperis* (sexual morph: *Nectria haematococca* f. sp. *piperis*) (Cuomo et al., 2007).

To overcome host defenses, *Fusarium graminearum* must have evolved an effective reserve in order to establish infection. In fungi, G protein coupled receptors (GPCRs) act as sensors for environmental cues on the cell membrane, which then activate the signaling pathways, and in *F. graminearum* 84 GPCR were predicted (Cuomo et al., 2007). Plant tissue penetration is a crucial step in the early stage of infection (Guo and Ma, 2014). The genome of *F. graminearum* is enriched with cutinase genes, which are responsible in cuticle degradation and penetration of the epidermis by the pathogen (Guo and Ma, 2014). The virulence of *F. graminearum* on both wheat and maize is determined by Lipase FGL1 (Cuomo et al., 2007). This lipase FGL1, along with MAPKGPMK1, is regulated by the RAS2 protein in *F*. *graminearum* (Bluhm et al., 2007). The process is clearly demonstrated by the MAPK cascade and signal RAS proteins (Bluhm et al., 2007). Mycotoxin DON is known to be the virulence factor in *F. graminearum* as the DON nonproducing strains do not show virulence or reduced virulence (Proctor et al., 1995). However, Tri5 mutants form appressoria-like structures similar to those of the wild-type strain (Boenisch and Schäfer, 2011). The production of DON depends on tissue types during the infection of wheat heads by *F. graminearum* (Guo and Ma, 2014). In addition, mutants that have increased sensitivity to environmental stresses such as oxidation, heavy metals, and antifungal compounds tend to have attenuated virulence as these mutants are likely to be vulnerable to the plant defense actions (Guo and Ma, 2014).

Results of studies on *Fusarium graminearum* shows how the fungus overcomes the host defense and establishes the disease. In highly diverse and highly competitive grassland populations, *F. graminearum* is well adapted to implement the disease by overcoming the host defense. Furthermore, *F. graminearum* shows high adaptability for environmental stresses.

### Bipolaris sorokiniana

**Figure 4:**
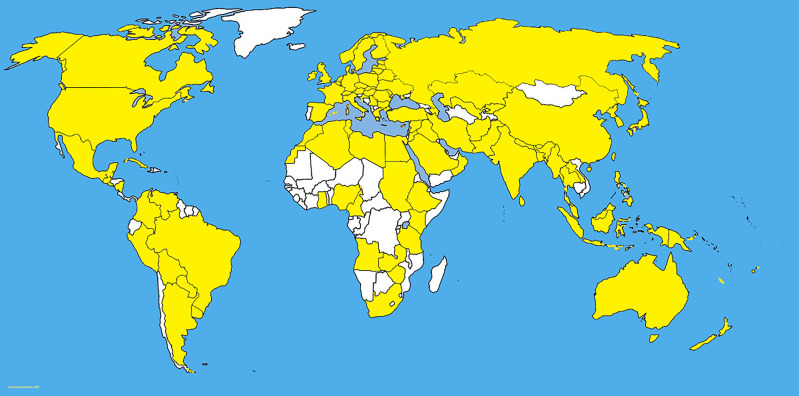
The dIstribution map of *Bipolaris sorokiniana*. The areas where infection have been reported are indicated in yellow (www.cabi.org/isc. Accessed on 25/03/2021).

**Figure 5:**
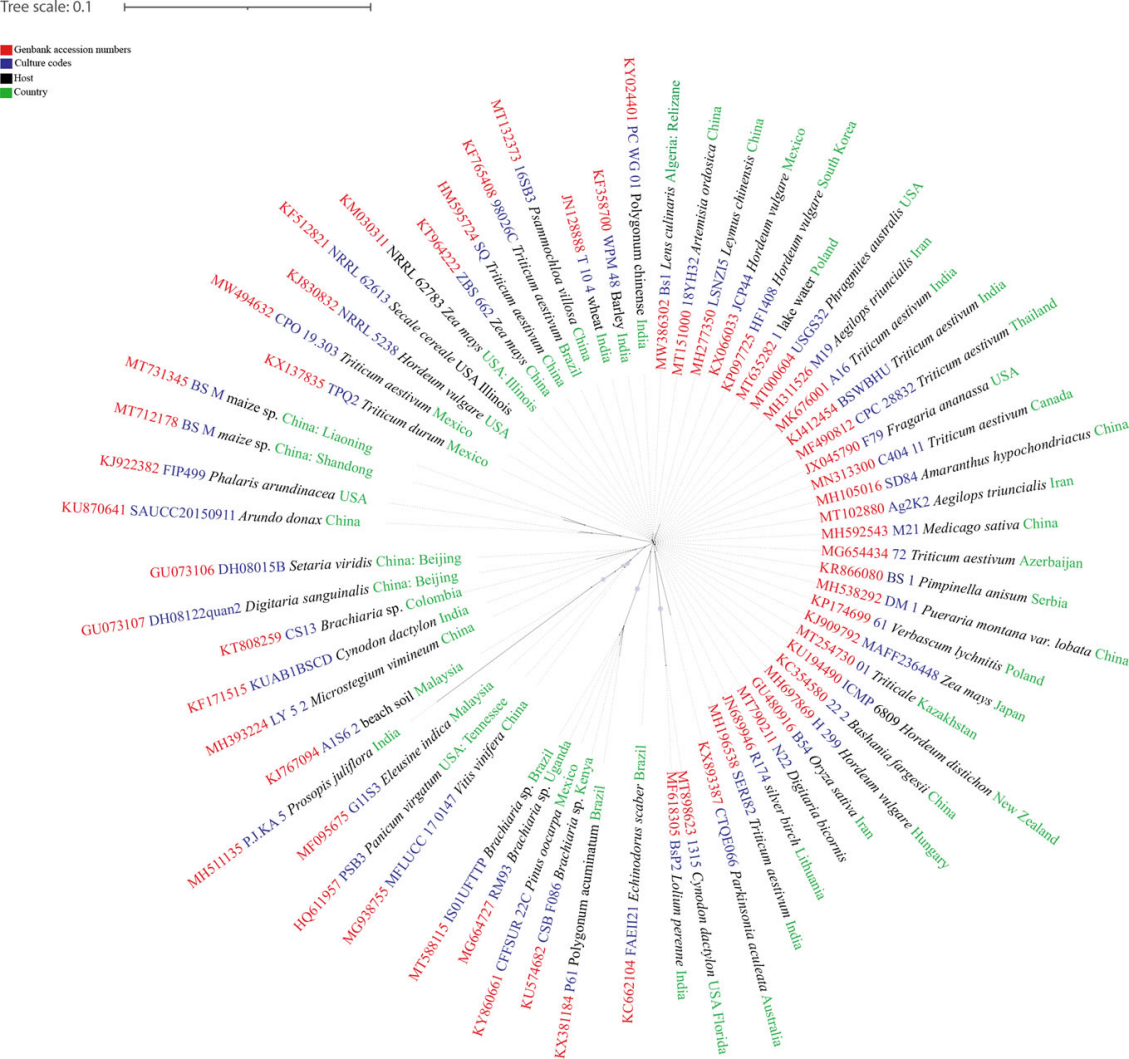
The unrooted RAxML bipartion unrooted phylogenetic tree of *Bipolaris sorokiniana* built from sequences retrieved from NCBI GenBank.

*Bipolaris* species are mostly found on grasses (Manamgoda et al., 2014; Bhunjun et al., 2020) and in this example we deal with *Bipolaris sorokiniana*. Although the population, pathogenicity, and DNA polymorphism of *Bipolaris sorokiniana* Shoemaker in natural grasslands have not been studied, but the behaviour of *B. sorokiniana* was well studied as a pathogen of economically important crops. Most recent studies on *B. sorokiniana* provides a good understanding about pathogenicity variations and DNA polymorphism of pathogenic fungal communities in a monoculture (Kang et al., 2021). (**Figures 4**, **5**) in diverse geographical areas. Several fungal pathogens are associated with wheat black points in different regions (Dai et al., 2011). *Bipolaris sorokiniana* shows a cosmopolitain distribution (Kang et al., 2021) with highly varied pathogenicities and genetic diversities (**Figures 4**, **5**) (**Table 1**). Aghemiri et al. (2015) has studied 46 samples of *B. sorokiniana* in Iran and it showed that *B. sorokiniana* grouped into highly distinct clusters representing different geographical regions. Hence, it is obvious that *B. sorokiniana* has higher adaptability towards different geographical conditions, which denotes that *B. sorokiniana* populations in different grassland communities might be varied with the physical conditions that the fungal community faces. In another study, 95 isolates of *B. sorokiniana* collected from various plant parts and cultivars of spring barley showed remarkable differences in pathogenicity (Baturo-Ciesniewska, 2011). Furthermore, no significant correlation among pathogenicity and origin of isolates was observed (Baturo-Ciesniewska, 2011). Population genetic variations among two different populations in two consecutive years were assesed by a most recent study of Kang et al. (2021). It revealed significant polymorphisms between isolates and proportional connectivity between geographic distance and genetic diversity. Moreover, the study revealed that the pathogenic variations in *B. sorokiniana* has no relevance to its geographical origins. Cross assays of *B. sorokiniana* among wheat and maize roots showed the ability of causing disease in both hosts. Clustering analyses of different isolates from the same area indicated the variability of genetic populations in *B. sorokiniana*. More interestingly, isolates from the same tissue sample showed higher variations. Leisovasvobodova et al. (2012) revealed that the limits of gene exchange were around 80–100 km, and beyond that gene exchange rates became very low. The studies relevant to the host infection are controversial. Yan et al. (2012) found that the systematic generation of infection is from root to leaf. However, Gyawali et al. (2012) revealed that isolates from root and leaf on the same host show differentiation of infections.

Studies of *Bipolaris sorokiniana* have revealed its higher adaptability to various environmental conditions. Even though all previous studies were carried out in monocultures, experiments such as those involving cross infection assays indicate that grassland comunities into some extent. *Bipolaris sorokiniana* can infect several grass species and has considerably variable infection mechanisms, which reveals the ability of retaining it successfully in natural grasslands. Hence, this reveals that *B. sorokiniana* in different grasslands have different pathogenicities and significantly different polymorphism.

### Colletotrichum graminicola

**Figure 6:**
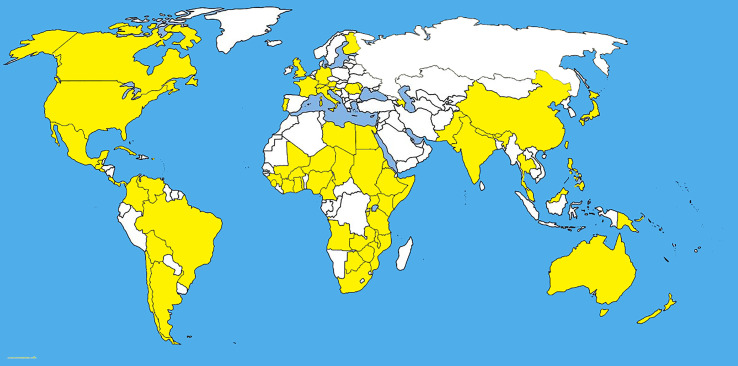
The distribution map of *Colletotrichum graminicola sensu lato.* The areas where infection have been reported are indicated in yellow (www.cabi.org/isc. Accessed on 25/03/2021).

*Colletotrichum* being a major pathogen causes diseases on grasses as well (Jayawardena et al., 2016; Bhunjun et al., 2021). *Colletotrichum graminicola* (Ces.) G. W. Wils. which is now considered to be in *Colletotrichum graminicola*-*caudatum* complex with 25 species (Bhunjun et al., 2021)*. Colletotrichum graminicola* (Ces.) G. W. Wils. causes anthracnose disease in maize, which causes stalk rot and seedling blight worldwide (Bergstrom and Nicholson, 1999) (**Figure 6**). Although, the effect of *C. graminicola* on roots is not well known, several studies have shown that the infection spreads through residues of soil and progresses to upper aerial sections (Leonard and Thompson, 1976; Keller and Bergstrom, 1988). The studies by Leonard and Thompson (1976), and Keller and Bergstrom (1988) are not based on molecular phylogeny. Hence it is not known the exact species they were dealing with, as the *C. graminicola* is a species complex. Based on ultrastructural studies, it is clear that anthracnose infection penetrates through the formation of appressorium with penetration peg (Eisermann et al., 2019).

According to the studies of Sunko et al. (2008), *Colletotrichum graminicola* shows morphological characteristics of root pathogens and spreads out to aerial plant organs. Nordzieke et al. (2019) studied infection stratergies of two different spore types of *C. graminicola.* Falcate conidia form in necrotic lesions and have to capability of forming appressoria. Hence, falcate conidia are key disease spreading propagules from plant to plant while oval conidia formed in vascular system of infected plants cause the distribution of disease within the plant. Furthermore, Nordzieke et al. (2019) showed that germinating falcate conidia are controlled by self-inhibitor mycosporine-glutamine, whereas it is absent in oval conidia. Moreover, studies revealed that oval conidia undergo germling fusions while falcate conidia do not. However, plant infection experiments revealed efficient leaf infections by oval conidia (Nordzieke et al., 2019). With such specifications on conidia, *C. graminicola* has the ability to infect various plant tissues, such as leaves, stems, and roots as well as grows saprophytically on dead plant material to over winter (Nordzieke et al., 2019).

At the onset of infection, *Colletotrichum graminicola* secretes proteins to avoid pathogen-associated molecular pattern recognition by the host (Eisermann et al., 2019). Eisermann et al. (2019) revealed that among the proteins secreted by *C. graminicola*, only a few are essential for fungal virulence. Among small clusters of *C. graminicola*, only a single protein is involved in infection process. There is a probable involvement of other proteins, even though it might not be able to detect experimentally. In the infection process, targeted deletion of 26 individual candidate genes and seven gene clusters with 32 genes of *C. graminicola* are known as a pathogenicity cluster (CLU5) of five co-linear genes. With the exception of CLU5b, encode secreted proteins CLU5a and CLU5d are required for full appressorial penetration.

Studies of *Colletotrichum graminicola* have demonstrated fungal adaptability on fungal infection, implementation of the disease and how the disease spreads through the plant. These are critical aspects in highly diverse grasslands. Current knowledge of *C. graminicola* is mainly based on monocultures and effects on infection mechanisms in highly diverse environments are not well known.

## Diseases of Humans and Other Animals

The natural grasslands are complex ecosystems of plants and animals (Gibson, 2009). Their interactions are shown to be highly variable (Gibson, 2009). Hence, the interactions between animals and grassland ecosystems cannot be neglected. Grasslands shelters an immense number of vertebrate and invertebrate animals and their interactions are vital for the grasslands (Gibson, 2009). Therefore, identification and understanding of fungal diseases on animals living in grasslands are important.

A direct study regarding fungal pathogens on grassland animals is not reported. Hence the linkage of current knowledge regarding domestic and industrial farms such as cattle farms can be taken to get an insight. A well-studied disease chronic facial eczema on sheep is caused by a grassland fungus *Pithomyces chartarum*. *Pithomyces chartarum* is a well-known saprobe in monocots including grasses (Laven et al., 2020; Munday et al., 2020) and it causes facial eczemas in ruminants and camelids (Laven et al., 2020). *Pithomyces chartarum* produces a mycotoxin known as sporidesmin which causes edema, ulceration and crusting dermatitis on face and ears in ruminants and camelids (Munday et al., 2020). Sporidesmin concentrates in bile and causes damages to liver through the bile duct (Laven et al., 2020).

Fungal diseases on humans relevant to grasslands have not studied directly and no published data is available on direct fungal infections on humans caused specifically by grassland fungi. But diseases caused by mycotoxins through the cattle industry was reported (van Egmond, 1983; Kemboi et al., 2020). Mycotoxins are hazardous to cattle as well as humans who consume dairy products (van Egmond, 1983; Kemboi et al., 2020). Main mycotoxin categories relevant to the dairy industry are Aflatoxins (AF) produced by *Aspergillus* species, deoxynivalenol (DON) produced by *Fusarium* species, fumonisins (FUM), ochratoxin A (OTA) produced by *Penicillium* and *Aspergillus* species, T-2 toxin (T-2) and zearalenone (ZEN) (Kemboi et al., 2020). The well-known aflatoxin M1 is a carcinogen (van Egmond, 1983; Kemboi et al., 2020).

Fungal pathogens of humans and other animals in grasslands are least studied, although, such pathogens are economically highly important. There should be emerging animal and human pathogens in grasslands around the world. Hence comprehensive studies on pathogens in grasslands are important.

## Conclusions

Natural grasslands are a vital component of several different types of terrestrial ecosystems but are not yet sufficiently studied (Gibson, 2009). Grasslands are highly complex ecosystems comprised of perennials and are dominated by members of the family Poaceae (Risser, 1988). With the high density of plants and the high density of below ground root systems, grasslands provide an ambient environment for microbial growth (Mommer et al., 2018; Ampt et al., 2019). The composition of the flora of the grasslands in various regions is affected by unique biological and ecological factors (Risser, 1988; Gibson, 2009). Hence, the interaction between the microbiota and their hosts becomes unique in each grassland system based on the floral composition.

There is a wide range of fungal lifestyles in nature, but our concern in this paper was focused on the pathogenic lifestyle of the fungi associated with natural grasslands. The fungal- plant interactions in grasslands can be described using two approaches—host specificity based and density dependent based. Research on fungal pathogens can be based on either phylogeny or pathogenicity (Janzen, 1970; Connell, 1971; Bever et al., 2015). Currently, there have been many taxonomy and phylogeny based studies on the fungi associated with grasses in different ecosystems, which are subject to change since many novel taxa are continuously being introduced. This situation provides the basis for developing an insight for resolving the taxonomic placement of identified and unidentified fungal taxa. In many cases these data cannot be used in accordance with the pathogenicity related studies unless the life mode is confirmed, specifically through the confirmation of the pathogenicity through the Koch postulate. Grasslands also cause disease of humans and other animals.

Grassland ecosystems are characterized by complex interactions between pathogens and their hosts. Although the majority of the grassland plants are monocots, there are dicots present among the monocots plants (Herben et al., 1993; Van Der and Sykes, 1993; Gibson, 2009). Furthermore, there is a high diversity of species in the family Poaceae. Hence, grasslands cannot be considered as a monoculture, and the interactions between monocots as well as between monocots and dicots need to be considered (Herben et al., 1993; Van Der and Sykes, 1993).

The fungal community below ground and above ground are highly diverse and contrasting. Furthermore, the distribution of fungal pathogens above and below ground in grasslands are complex (Van Ruijven et al., 2003; Van Ruijven and Berendse, 2005; Van Ruijven and Berendse, 2009; Cong et al., 2014; Cappelli et al., 2020). Comparatively speaking, the ecology-based studies of fungal pathogens below ground are much more common than the ecology based studies of fungi above ground in natural grasslands. Studies of the fungal pathogens of members of the Poaceae are mainly focused on agricultural monocultures. Hence, there is a dearth of information on above ground fungal pathogens rather than those below ground. In certain cases, these data are applicable to natural grasslands as far as the same species is concerned. However, the effects of complex ecological intereactions in the natural grasslands cannot be neglected.

Fungal pathogens of animals in grasslands are poorly studied. Natural grasslands provide grazing sites for large ruminants (Gibson, 2009). In many instances those grazing ruminants directly or indirectly involve human activities and the economy. Hence it is important to study relationships between grassland animals and fungi. Chronic facial eczema caused by *Pithomyces chartarum* shows how important it is to understand fungal communities in grasslands (Laven et al., 2020; Munday et al., 2020). In a way, grasslands can be a reservoir of fungal pathogens.

In this review, we have provided four examples where a complete study has been carried out on the pathogenicity and genomics of a particular pathogen. They are based on agricultural monocultures with the help of natural grassland based data. There is a necessity of exploring the natural grasslands to identify novel fungal species. In the meantime, confirming the pathogenicity is also very important. Agronomically, it is important to study the pathogenic community in grasslands as a reservoir of fungal pathogens and understanding the dynamics of the fungal community further helps to understand the effects of fungal pathogens in agricultural multi-lines. The study of *Pyrenophora tritici-repentis* demonstrated just how important it is to perform a thorough study even on a well-known pathogen, since there are many races with different life modes and attributes. The behavior of the pathogen in natural grassland is also important to observe in order to understand changes in gene expression and pathogenicity.

Plant diversity of grasslands is highly variable around the world. However, the stable natural grasslands are rich in host diversity. The belowground host components shapes the total fungal community and the above ground fungal community highly affects the productivity and the biomass of the grassland. Hence, the stability of grasslands is highly dependent upon the balanced interactions of those factors together with other biotic and abiotic factors. The behavior of specialist pathogens and generalist pathogens in grasslands is crucial for the stability of these communities. Heterospecific neighbors in grasslands disturbs the spread of specialist pathogens but also could facilitate the spread of generalist pathogens. However, fungal disease spread in grasslands depend on huge number of biotic and abiotic factors. Different fungal pathogens act differently on the hosts. Meantime, host respond is highly variable. Studies of *Pyrenophora tritici-repentis* on grasses provide good insight on how pathogenic response of different host genotypes vary towards the same pathogen. In addition, different hosts or genotypes of grasses can induce host-fungal pathogens without causing symptoms. Hence, in a way, grasslands can act as a reservoir for fungal pathogens. Even though pathogenic mechanisms of fungi have been well explained from monocultures, the virulence of the fungus varies with the high diversity in grass lands. Many factors such as heterospecific neighbors, tightly and closely arranged roots, effects of root exudates and root microbiomes, and different host traits can control disease spread.

The knowledge of host-pathogen interactions in grasslands can be used for agricultural purposes. Strategic grasslands can be used among the crop fields to control the spread of diseases. Grasslands in between crop fields can act as dense heterospecific host sites for many pathogens and this can immensely reduce the disease spread among the adjusent crop fields. Furthermore, the presence of highly diverse grasslands can reduce the disease insidence of the specialist pathogens. Grass heterospecific neighbor effects of the soil-borne fungal pathogens can be explained in two ways. First, neighbor effects though plant traits or root exudates. Second, neighbor effects through root microbiome. However the loss of dominant plant species in grasslands can increase the extent of disease in the system (Mitchell et al., 2002). Hence, proper understanding of the dominant species in the strategic grasslands and maintainance of the dominant species is important for disease control.

Grasslands are an ecologically and economically important component of the earth’s vegitation. Fungal communities in grasslands play a huge role on the stability of grasslands. Having more complete knowledge of fungal pathogens in grass lands is important for developing an understanding of grassland ecology. In addition, understanding behaviour of fungal pathogens in highly diverse grasslands may provide novel insights towards being able to control the diseases in commercial crop fields. In this review, we address the effects of fungal pathogens in grasslands and discussed their complex interactions.

## Author Contributions

AK, ST, KH and SCK designed the review. SL, JK and SK provided the grant. AK wrote the manuscript with ST, CN, SCK and SLS. RJ, SA, JX, KH, JK and SL reviewed and edited the manuscript. All authors reviewed and approved the final manuscript. All authors contributed to the article and approved the submitted version.

## Funding

International Postdoctoral Exchange Fellowship Program (number Y9180822S1), CAS President’s International Fellowship Initiative (PIFI) number 2020PC0009 and young staff under the grant number: 2020FYC0002, China Postdoctoral Science Foundation, National Science Foundation of China (NSFC) under the project codes 31850410488 and 31851110759, the Yunnan Human Resources, and Security Department Foundation and Thailand Research Funds for the grant “Impact of climate change on fungal diversity and biogeography in the Greater Mekong Subregion (RDG6130001)” are thanked for funding. This research work was partially supported by Chiang Mai University.

## Conflict of Interest

The authors declare that the research was conducted in the absence of any commercial or financial relationships that could be construed as a potential conflict of interest.

## Publisher’s Note

All claims expressed in this article are solely those of the authors and do not necessarily represent those of their affiliated organizations, or those of the publisher, the editors and the reviewers. Any product that may be evaluated in this article, or claim that may be made by its manufacturer, is not guaranteed or endorsed by the publisher.
